# Subjective time under altered states of consciousness in ayahuasca users in shamanistic rituals involving music

**DOI:** 10.1590/1414-431X20209278

**Published:** 2020-06-19

**Authors:** A.P.S. Campagnoli, L.A.S. Pereira, J.L.O. Bueno

**Affiliations:** Departamento de Psicologia, Faculdade de Filosofia, Ciências e Letras de Ribeirão Preto, Universidade de São Paulo, Ribeirão Preto, SP, Brasil

**Keywords:** Subjective time, Time, Ayahuasca, Hallucinogen, Altered states of consciousness, Music

## Abstract

Ayahuasca is described as a hallucinogenic substance whose property is to alter the subjective experience of time and impair the perception of the passage of time during stimuli of more than two to three seconds. The dose-dependent effects of two concentrations of ayahuasca in the ritualistic context were investigated employing temporal reproduction tasks in participants experienced in shamanistic ayahuasca rituals. The study was conducted on nine healthy volunteers who ingested two doses of ayahuasca at two times during a ritual session. The doses of each session, consumed in amounts ranging from 20 to 60 mL, were either of low concentration or of experimental ayahuasca according to a double-blind procedure. Participants performed the task of immediately listening and reproducing, with a laptop, 20-s musical stimuli during the session. The results showed that significant temporal distortion was triggered by the musical stimulus presented without the ingestion of ayahuasca, with means of 16.33 to 16.52 s. There were minor temporal distortions after ingestion of ayahuasca: a mean of 17.91 s for control ayahuasca and of 18.38 s for experimental ayahuasca. These results with less temporal distortion among participants with ayahuasca intake disagree with other studies of hallucinogens involving temporal reproduction.

## Introduction

Natural substances extracted and developed from plants of the Amazon rainforest by the native people have been a focus of studies aimed at understanding the effects of altered states of consciousness on human psychological processes. These altered states of consciousness can be assessed by recording subjective time changes among participants in shamanistic rituals, not only in indigenous villages, but also in rituals practiced in the rural and urban context.

The ayahuasca beverage is an example of such substances. In Brazil, ayahuasca has legal status in religious and scientific contexts when prepared by cooking the leaves of the *Psychotria viridis* shrub with the *Banisteriopsis caapi* vine (1). There are differences in the concentrations and proportions of alkaloids found in ayahuasca related to the preparation method (2).

*Psychotria viridis* contains N,N-dimethyltryptamine (DMT), which is inactivated by monoamine oxidase inhibitors (MAO-A) in the liver when administered orally through first pass metabolism; *Banisteriopsis caapi* contains β-carbolines, reversible monoamine oxidase inhibitors (MAOI) (2). Inhibition of MAO can raise levels of serotonin, noradrenaline, and dopamine in the brain. The β-carbolines in ayahuasca act by inhibiting MAO, causing oral DMT to be active and reach the systemic circulation and the central nervous system, promoting new perceptions of reality such as mental images (3,4). The psychoactive effects of ayahuasca start 30 to 60 min after ingestion, reaching a maximum intensity between 60 and 120 min, and can last up to four hours after ingestion (5).

Schultes et al. (6) have described ayahuasca as a hallucinogenic beverage containing the hallucinogen DMT and β-carboline alkaloids such as harmine, tetrahydroharmine, and harmaline as active ingredients. To date, the literature considers ayahuasca as a hallucinogen, involved in the regulation of mood, memory, emotions, and perceptions similar to LSD (lyserg-saure-diathylamid) and psilocybin (*Psilocybe cubensis*) (7), that acts on frontal and limbic areas of the brain, rich in serotonin-2A (5-HT_2A_) receptor. The anxiolytic potential of ayahuasca appears to be related to the agonist activity of DMT on 5-HT_2A_ cortical receptors. Several studies, however, have discussed the limitations of attributing hallucinogenic properties to certain preparations that alter states of consciousness (7–9).

The psychoactive effect of ayahuasca on the brain has been found to involve changes in neural circuits recorded by an electroencephalogram apparatus (10–14). Effects of chronic intermittent exposure to ayahuasca in mice have been explored (15). In addition, in terms of therapeutic purposes, ayahuasca has shown psychological and physiologic effects, with potential use in mental illness as a substance with antidepressant potential (4,14,16). Although ayahuasca has been widely studied within a pharmacological context (2), few studies have examined its properties using an ecological approach, such as in the ritual context. Studies have examined how the environment influences the state of the participants and investigated how the religious content involved in these rituals results in altered states of consciousness (17).

Moreira and MacRae (17) report that, according to the organizers of ceremonies with ayahuasca, two conditions are extremely necessary for the rituals based on the use of this drink: the ritual context and the songs performed during the ceremony.

Altered states of consciousness correspond to a qualitative change in the global pattern of mental functioning (18). Mabit (19) points out that this alteration of consciousness aroused by ayahuasca does not trigger a loss of consciousness, but rather a perceptual change. Shanon (20) proposes that consciousness is a group of criteria that define values, determining how man experiences the world, mentally and physically. Thus, substances such as ayahuasca represent a viable empirical basis for the biological investigation of altered states of consciousness and provide adequate experimental models to approach concepts and elaborate hypotheses about the temporal functioning and representation of internal time (9). Subjective time studies are a form of access to internal states (21) and, so far, we have not found studies investigating the effect of altered state of consciousness with ayahuasca in the ritualistic context on the subjective perception of time in the specialized literature.

The subjective perception of time is essential for the perception of reality, and the processing of temporal information is essential for everyday life (22–24). Subjective time is the relative estimation of a certain period of time, that is, the duration of the intervals between two successive events (25).

The New Experimental Aesthetic proposed by Berlyne (26) has used artistic works to investigate subjective time, as well as the effects of the characteristics of these works on some aspect of the behavior of organisms. The characteristics of a musical composition can generate temporal distortions in the listener. Many researchers have studied subjective time with the use of musical stimuli (27–29).

Among a wide diversity of models proposed to explain subjective time, some have been especially used for the discussion of subjective time perception of esthetic stimuli such as the storage size model (8), the attentional model (30), the contextual change model (31), and the contrast or expectation model (29).

Other models such as sensory processing require that a repetitive and cumulative mechanism be stored in the form of pulses by a device that would generate internal signals of time, acting as a “time organ” named “internal clock” (32).

Temporal distortions can also be observed during the use of substances that have a hallucinogenic effect. According to Shanon (33), altered states of consciousness, in general and in the ayahuasca experience, can not only distort subjective time, but also cause a rupture in temporal perception and refer to a sense of timelessness. Mitrani et al. (34) employed LSD and mescaline hallucinogens in a study that investigated time intervals between 300 and 1000 ms in a task of identifying the duration within short time intervals and showed that LSD and mescaline did not affect the performance of the participants, although all subjects reported the loss of sense of time in the course of the experiment. Wittmann et al. (7) showed that psilocybin significantly impaired the ability of the participants to reproduce intervals lasting longer than 2.5 s. These effects were accompanied by deficits in working memory, subjective changes in the conscious state, and disturbances in the sense of time. The results of the study suggested that the serotonin system is selectively involved in processing intervals of two to three seconds and in voluntary control of the speed of movement.

The objective of the present study was to investigate the effects of listening to musical stimuli on subjective time, as a function of two ayahuasca concentrations, on participants experienced with ayahuasca ritual practice during the shamanic ritual in the urban context.

## Material and Methods

### Participants

The participants consisted of 15 Brazilian persons (eight women and seven men), mean age of 35.6 years, with self-reported normal hearing. People with experience in shamanistic ritual practices were recruited for the experiment, with those who had ingested ayahuasca beverage more than 60 times in the last three years being considered experienced. Criteria for psychological and medical restrictions were used to exclude participants: current use of any psychiatric medication, personal history of psychiatric illness, any neurologic disorder or brain injury in the past and cardiovascular disease. Participants who used tobacco and/or caffeine on a regular basis were required not to consume either substance at least one hour before the ritual. Participants who used ethyl alcohol on a daily basis were asked to abstain for 24 h before participating in the study. The study was approved by the Research Ethics Committee of the Ribeirão Preto School of Philosophy, Sciences and Literature of the University of São Paulo, Ribeirão Preto Campus, Brazil (protocol No. 1.778.007) and all subjects gave written informed consent to participate.

### Material and equipment

#### Musical stimuli

Thirty musical pieces were used, each lasting 20 s, with the same thematic identity of the songs played in the rituals of the Institute. The musical stimuli were constructed in a recording studio of the Center for Experimental Aesthetics, Universidade de São Paulo at Ribeirão Preto. The following equipment was used for audio treatment: an M-AUDIO 61es keystation, an iMac 20" computer, an M-AUDIO Firewire 1814 sound card, a pair of M-AUDIO model BX8a audio monitors, a Ciclotron CGE 2312s equalizer, a Behringer channel distributor, model Powerplay PRO-XL, an M-AUDIO condenser microphone, Luna model with a shelf, P2-P10 and P10-P10 Santo Angelo M30 balanced cables, and a Sony stereo system, model HCD-GT444.

### Preparation of experimental and control ayahuasca

The ayahuasca, referred to in this study as “experimental ayahuasca”, was the ayahuasca normally used by the institute in its rituals and was prepared with the monitoring of the institute's overseer (a person with experience in producing ayahuasca). A decoction of 60 kg of the vine *Banisteriopsis caapi* (tucunacá variety) with 15 kg of leaves of the shrub *Psychotria viridis* was used for the preparation of ayahuasca. This mixture was divided into three pots, two thirds of which were divided into two pots, each containing 40 L of water. The material was first boiled for four hours and the volume obtained was reduced to 20 L of beverage per pot. The beverage was strained to separate the vine and leaves and reserved. The same boiling procedure was carried out two more times, resulting in the production of 120 L of beverage. New amounts of vine stems and leaves, separated from the initial two-thirds, were added to the 120 L of beverage, and boiled for four hours, with the volume being reduced to 60 L of beverage. The resulting infusion was separated from the vine stems and leaves and submitted to a final boil, reaching the concentration of 30 L desired by the supervisor and representing the experimental ayahuasca.

The control ayahuasca was the result of the infusion of the first boiling process in the preparation of experimental ayahuasca.

All ayahuasca used in the study came from the same production batch.

The chromatographic analyses of ayahuasca were performed using the “LC-LC-QqToFMS” Multiuser equipment [FAPESP (process no. 2004/09498-2), under the responsibility of G.M. Titato, Laboratory of Chromatography, Institute of Chemistry of São Carlos, USP, Brazil]. The compounds were detected with a high-resolution quadrupole/time-of-flight type mass detector (TOF/MS) model MICROTOF-QII (Bruker Daltonics, USA). The compounds under study were ionized by electrospray in the positive ion mode under the following operating conditions: capillary voltage (4.5 kV); nebulizer gas pressure (4 bar); drying gas flow rate (8 L/min.); monitored mass band (100–3000 Daltons); spectrum acquisition rate (1 Hz). The results obtained are described in Table 1.

**Table 1:** Comparison between the control and experimental concentrations of ayahuasca, showing the percentage of the area (by the intensity of the peak of the chromatogram) of each compound (N,N-dimethyltryptamine (DMT), harmine, harmaline, tetrahydroharmine) and the proportion of harmine, harmaline, and tetrahydroharmine in relation to DMT.

	Control	Experimental
DMT (area %)	14.2	7.4
Harmine (area %)	44.4	73.4
Harmaline (area %)	9.6	4.9
Tetrahydroharmine (area %)	31.8	14.3
Harmine / DMT	3.12	9.91
Harmaline / DMT	0.67	0.66
Tetrahydroharmine / DMT	2.24	1.93

The proportion of harmin, harmaline, and tetrahydroharmine (Table 1) to the amount of DMT indicated (considering the control ayahuasca sample as having the least effect and the experimental ayahuasca sample as having the greatest psychoactive effect) that the greater the harmine/DMT ratio, the greater the effect of the tea; the harmaline/DMT ratio had little influence on the effect and the tetrahydroharmine/DMT ratio seemed to have an inverse effect, i.e., the lower the ratio, the greater the effect of the tea.

Twenty tempered glass containers were constructed, with opaque black glassware paint, of cylindrical tubular shape, 30 mm in diameter and 160 mm high, with flat bottoms and top openings with a thicker edge. The containers were used to administer a volume of up to 60 mL to the participants, minimizing the perception of the differences in the two ayahuasca teas since both liquids had the same smell and flavor, but different consistencies and colorations due to different concentrations.

#### Experimental control equipment and data logging

An IBM-PC laptop with the experimental program Wav Surfer (35), implemented in Visual Basic 6.0 for Windows was used to monitor the tasks, to store and play musical excerpts and beeps, and to record temporal reproductions. A color keypad was adapted from a numeric keypad coupled to the laptop to play the music tracks and to perform the temporal playback task: the “enter” key was colored in blue and marked with “*escutar*” (listen) to start the musical excerpts; the minus key (-), in green color, was marked with “*início*” (start), and the plus key (+), in red color, was marked with “*fim*” (end) to perform the time task. A closed JBL J55i headset was used to listen to the musical excerpts. A beep (50 ms) was provided using the free sound synthesis Csound 4.19 software (sample rate: 44.1 kHz; bandwidth: 16 bit; mode: mono).

### Procedures

The study was conducted from March 2017 to June 2017 in the state of São Paulo, Brazil, in an urban area where there are Institutes that use ayahuasca.

The ritual sessions were held in an appropriate place for performing shamanic rituals, located in a circular area with 22.5 m in diameter with an 11-m high main center, during the period from 5 to 8 pm. On the first day of the session, before the beginning of data collection, all participants went through training in order to learn the task of reproducing the time of the musical stimuli.

Participants went through ritual sessions, each consisting of the following sequence of activities: participants were asked to sit around a campfire and instructions were verbally presented; the first time estimation task was performed; the participants consumed ayahuasca; the lights were turned off and the 90-min ritual began, during which shamanic songs were continuously presented [see (36) to listen to unpublished compositions of some songs used in the sessions] and participants could freely circulate around the Institute area; the music was interrupted, the lights were turned on and the second time estimation task was performed; the lights were turned off; the second part of the ritual was 90 min long, during which shamanic songs were continuously presented; the music was interrupted, the lights were turned on, and the third time estimation task was performed; the ritual was completed with brief words from the leader.

Ayahuasca could be consumed with the experimental or the control concentration, depending on the scheduled session. The amount consumed ranged from 20 to 60 mL, depending on the participant's wishes. On each ritual day, participants were divided into two groups, one ingesting experimental ayahuasca and the other ingesting control ayahuasca. Each participant was submitted to four sessions, two rituals with experimental ayahuasca and two rituals with control ayahuasca. Data were collected on five days of sessions, with each session being attended by 10 to 15 participants. The interval between session days was variable, and could be up to 20 days. The researchers did not use ayahuasca during the research rituals.

For the task of subjective time evaluation, the participants were asked to sit on chairs around tables in front of the altar. The participant used a headset to listen to 20 s of music stimulation using the numeric keypad on the table and clicking the appropriate key for stimulus reproduction. Immediately after each presentation, the participant was asked to estimate the duration of the musical excerpt using the appropriate key of the numeric keypad, one key to start and another to end the reproduction period signaled by a beep for feedback from the participants regarding the timing of reproduction. In each temporal reproduction task, the participants listened to and reproduced two of the 30 musical stimuli, each 20 s long, which were previously selected and presented at random, with no repetition of stimuli in any phase or on any session day.

### Statistical analysis

The participants remained conscious and lucid during the rituals while data were being collected. Subjects who experienced vomiting and/or did not ingest the second dose of ayahuasca continued to participate in the ritual. Six participants were excluded because they missed one or more of the four sessions. The final sample consisted of nine participants (five women and four men, mean age 37.7 years) five of whom reported never having used other psychoactive substances for altered consciousness; three reported discontinuing the use of other psychoactive substances for more than four years, and one participant reported making occasional use of *Cannabis sativa*.

Statistical analyses were used to test the effect of ayahuasca and of its concentration on temporal reproduction. The data of the middle and final conditions of the ritual were grouped and were denominated as “conditions during the ritual”, since no statistically significant difference was found between them. Repeated measures analysis of variance with two trial factors (ANOVA) was used for comparison between the means of temporal reproduction of the participants before ingestion and the means after ingestion of ayahuasca; data from the control and experimental ayahuasca conditions were grouped. Student's *t*-test for paired samples was applied to compare the mean values of the temporal reproductions and the real value of the musical stimuli obtained in each condition before the rituals and during the rituals for experimental ayahuasca and control. The level of significance was set at P≤0.05 in all analyses.

## Results

The means of temporal reproduction of the participants before and after ingestion of ayahuasca were compared with the grouping of data from the control and experimental ayahuasca. Values prior to ingestion (16.425 s) were significantly lower than values after ingestion (18.149 s), (F_1,8_=9.03, P=0.017).

**Figure 1:**
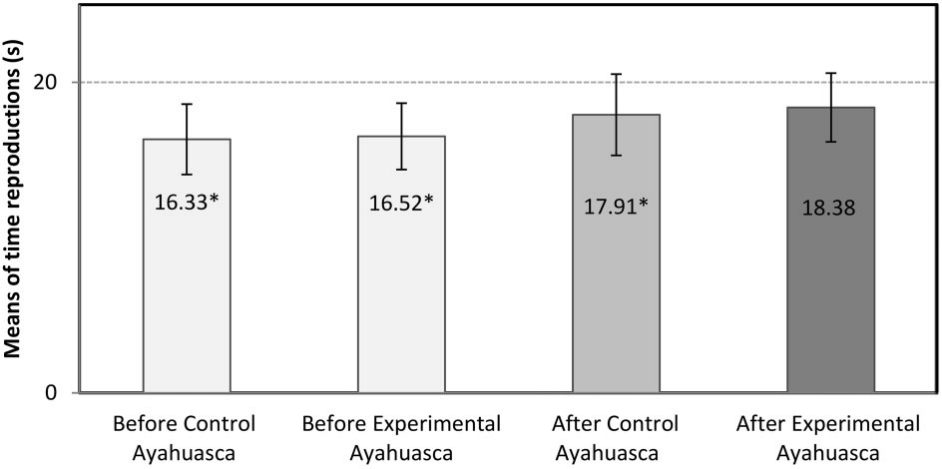
Means and standard deviations of time reproductions before and after control and experimental ayahuasca conditions. *P<0.05 compared to the 20 s musical stimuli (dotted line) (Student's *t*-test).

Figure 1 shows the comparison between the mean values of the temporal reproductions and the real value of the musical stimuli obtained in the conditions before the rituals and during the rituals for experimental ayahuasca and control. There was a significant difference in the temporal distortions in the conditions before the ritual without experimental ayahuasca (*t*_8_=–4.886, P=0.001), before the ritual without control ayahuasca (*t*_8_=–4.856, P=0.001), and during the ritual with control ayahuasca (*t*_8_=–2.389, P=0.044). All distortions were underestimated, that is, values were lower than the real time of the stimuli.

## Discussion

Our study showed that there was no temporal distortion obtained with the use of ayahuasca because it is a classic hallucinogen. Ayahuasca is described as a hallucinogenic beverage containing DMT and β-carbolines alkaloids such as harmine, tetrahydroharmine, and harmaline as active ingredients (6). Shulgin and Shulgin (3), although accepting that β-carbolines may have some psychoactive effect and contribute to the psychotropic activity of ayahuasca, were uncertain about the psychotropic properties of β-carbolines as “hallucinogenic” or “psychedelic”. Nichols (37) stated that the term hallucinogen was originally coined to designate substances that produce hallucinations, an effect, however, that they do not ordinarily elicit, at least at typical dosages. Thus, that name is a misnomer.

The musical stimulus experienced immediately before the ingestion of ayahuasca caused distortions with responses of temporal underestimation. Studies about timing in individuals without altered consciousness have shown that the duration of temporal intervals in the temporal reproduction task are estimated more accurately at intervals of approximately two to three seconds, while longer intervals are substantially underestimated (38). When the time estimate involves motor control for the stimulus reproduction task, as in this study, the ability to synchronize accurately becomes weaker for stimuli lasting longer than two seconds (39).

The participants consuming a higher dose of ayahuasca concentration (experimental ayahuasca) showed less temporal underestimation of the duration of the musical stimulus than the participants consuming a lower dose (control). In addition, the results of the participants using the experimental concentration did not show significant differences between the estimated and the actual duration of the musical stimulus, indicating a greater precision in temporal reproduction.

The accuracy of the experimental ayahuasca time reproduction task may be related to the increase in the level of dopamine in the brain. The β-carbolines present in ayahuasca inhibit MAO, raising levels of neurotransmitters such as dopamine. The dopaminergic systems are considered to be closely related to the processing of subjective time, and dopamine is also a fundamental neurotransmitter for the control of movements. Several neurotransmitter systems such as the serotonergic ones also play a role in temporal processing within seconds (7).

The present data showing that the use of experimental ayahuasca was accompanied by more precise temporal evaluations than the use of the control ayahuasca did not support the idea that ayahuasca acts as a hallucinogenic substance, as suggested by several studies reporting that hallucinogens cause strong alterations in temporal perception (7–9,34). It is possible that the duration of the music experience as part of the ritual also affected the results. However, this possibility was not examined systematically with adequate control in this study, suggesting new possibilities for analysis in future studies.

The amount of DMT in the control ayahuasca was found to be higher than the amount of DMT in the experimental ayahuasca. However, chromatography analysis showed that the amount of harmine contained in the experimental ayahuasca was greater than that in the control ayahuasca. The boiling time for the ayahuasca production process in this study degraded a large part of DMT present in leaves (*Psychotria viridis*) and concentrated the harmine contained in the vine (*Banisteriopsis caapi*). The experimental ayahuasca is the one used in a ritualistic context, which goes through four long boiling processes, with twice as many leaves and vines used compared to the control ayahuasca, for which only one boiling step and one quantity of leaves and vines were used. Thus, the experimental ayahuasca had a greater psychoactive effect than the control ayahuasca (G.M. Titatto, Laboratory of Chromatography, Institute of Chemistry of São Carlos, USP, Brazil, oral communication).

Thus, the present results suggested that the action of ayahuasca depends on the substances involved in its preparation and on the concentration ingested by the participants. Similarly, the effects of different psychoactive drugs may also be different (6,7,9,10).

Studies of brain electrical activity with electroencephalogram (EEG) during the action of hallucinogens help us to understand the psychoactive properties of ayahuasca and its possible relationship with hallucinogenic effects. Experiments outside the ritual context that record spontaneous electrical activity in the brain indicate that most psychedelic compounds tend to reduce slow wave activity (alpha and theta) and increase fast wave activity (beta) (10–14). Don et al. (11), analyzing the effect of a dose of ayahuasca during ritualistic use, transferred their participants to an adjacent room to perform EEGs and found a more activated pattern of electrical activity, with statistically significant increases in the beta band from 14 to 30 Hz and a tendency to a decrease in the power of the slow (theta and alpha) brain rhythms after the ingestion of a dose of ayahuasca. On the other hand, Hoffmann et al. (12 [Bibr B13]), analyzing the effects of three doses of ayahuasca at the place of the ritual with EEG measurements at the end of the ritual, reported an increase in the alpha and theta waves and unchanged beta activity. The authors concluded that ayahuasca appears to have different effects on brain functions than other traditional psychedelics.

The role of context has been emphasized in studies on the effects of drug use (40). There is an important influence of the environment on altered states of consciousness, especially on the shamanic journey. Several authors have pointed out that, in order for ayahuasca to produce its effects, the context of the ritual and the songs performed during the ceremony are essential (17).

In the present study, participants had experience with ayahuasca in the ritualistic context and data collection became an integral part of the ritual, with two doses of ayahuasca and data collected during and at the end of the ritual, which may produce more reliable data of the psychological properties of ayahuasca. This context is different from other studies performed in a laboratory environment, outside the ritual or with participants who were experienced users of only hallucinogens (10–14).

The increase in the alpha wave that occurs under the effect of ayahuasca in a ritualistic context is associated with an attentional gain of the altered state (12). The results suggested a relationship between the greater precision in temporal reproduction with ayahuasca as a function of the beverage concentration and the greater attention demanded by the temporal estimation in the context of ritual consumption.

Many of the studies conducted on ayahuasca generally use participants who currently use one or more substances such as psilocybin, LSD, ketamine, peyote, mescaline, *Cannabis sativa*, cocaine, MDMA, or amphetamines. These other substances occupy neural circuits similar to those of ayahuasca. Concomitant intake of these substances can interfere with the specific effect of ayahuasca (37), affecting the participants' cognitive processing.

The most recent literature review did not show another study that systematically assessed the impact of ayahuasca on temporal processing. Our main objective was to clarify whether ayahuasca, at the typical concentration used in a ritualistic context, induced specific effects on temporal control involving listening to musical stimuli. We concluded that the results of the present study suggested that the action of ayahuasca depended on its preparation and the concentration used, since the temporal distortions varied between experimental ayahuasca, control ayahuasca, and before and after ingestion. The study also highlighted the importance of the context of the shamanic ritual, since most studies on the effect of ayahuasca report data collected in the laboratory. Experimental access to altered states of consciousness under the effect of ayahuasca may have been facilitated by recording different measures of effects and data collection conditions, since the subjective time distortion measure was used in a task of musical stimulus reproduction by experienced participants using only ayahuasca. This study also showed that altered states of consciousness with ayahuasca depended on factors other than pharmacological ones, expanding the literature on the role of the ritual context.

The confirmation that ayahuasca has no hallucinogenic effects because it does not produce significant temporal distortions has consequences about the understanding of the perception of reality, since the subjective perception of time is essential for the perception of reality (22–24). Hallucinogens produce strong changes in the perception of time, a sense of delayed passage of time, and a subjective overestimation of time intervals (7).

Although the sample of the present study was small, these results may be relevant to the understanding of the properties of ayahuasca in the ritual context, and future studies using a larger number of participants, more concentrated doses, and other substances used in shamanic rituals could increase knowledge on this subject.
